# Supplementary material for: Clinical and Immunologic Characteristics of Colorectal Cancer Tumors Expressing LY6G6D
Source: Int J Mol Sci. 2024 May 14;25(10):5345. doi: 10.3390/ijms25105345 (PMC11121234; doi:10.3390/ijms25105345)
Supplement: Supplementary file 1 [file ijms-25-05345-s001.zip › ijms-2980723-supplementary.pdf]

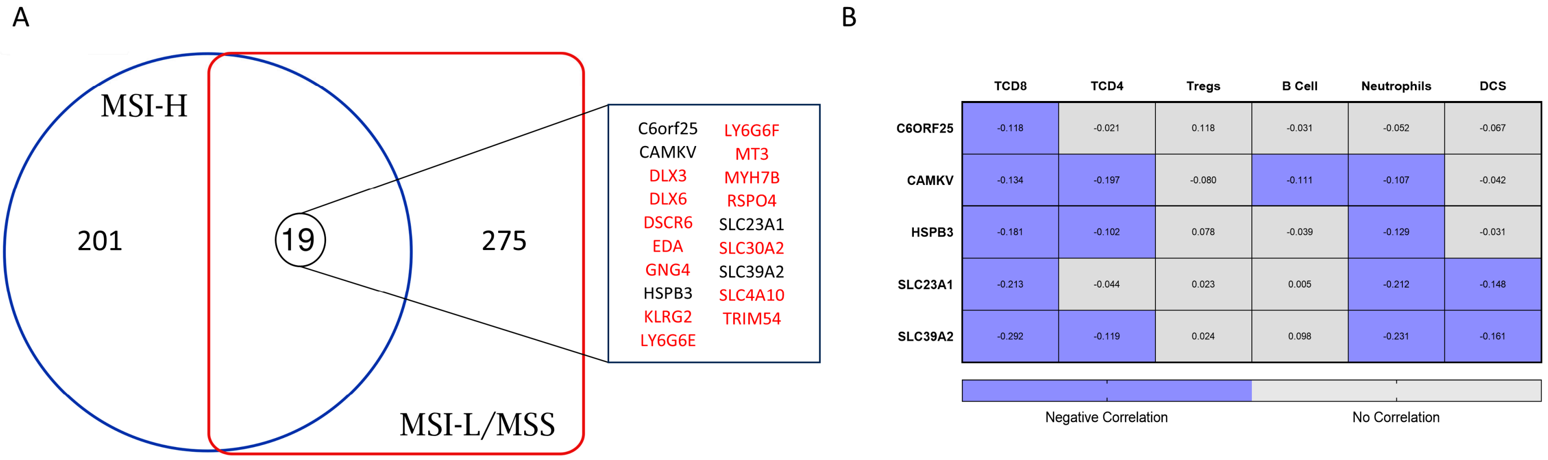

Supplementary Figure 1. Transcriptomic profiling of CRC tumors with high expression of LY6G6D. a) Venn-diagram with the genes on each set (MSI-H and MSI-L/MSS). b) Immune association in CRC patients of the expression of the selected genes with some of the more relevant immune populations related to cancer.
